# Gene Expression by a Model Fungus in the Ascomycota Provides Insight Into the Decay of Fungal Necromass

**DOI:** 10.1111/1462-2920.70006

**Published:** 2024-12-08

**Authors:** Irshad Ul Haq, Peter Kennedy, Kathryn M. Schreiner, Julia C. Agnich, Jonathan S. Schilling

**Affiliations:** ^1^ Department of Plant and Microbial Biology, College of Biological Sciences University of Minnesota Twin Cities Minnesota USA; ^2^ Department of Chemistry and Biochemistry University of Minnesota Duluth Duluth Minnesota USA; ^3^ Large Lakes Observatory University of Minnesota Duluth Duluth Minnesota USA

**Keywords:** CAZyme, chitinase, glucanase, *Hyaloscypha bicolor*, melanin, necromass, protease, *Trichoderma reesei*

## Abstract

Dead fungal cells, known as necromass, are increasingly recognised as significant contributors to long‐term soil carbon pools, yet the genes involved in necromass decomposition are poorly understood. In particular, how microorganisms degrade necromass with differing initial cell wall chemical compositions using carbohydrate‐active enzymes (CAZymes) has not been well studied. Based on the frequent occurrence and high abundance of the fungal genus *Trichoderma* on decaying fungal necromass in situ, we grew *Trichoderma reesei* RUT‐C30 on low and high melanin necromass of *Hyaloscypha bicolor* (Ascomycota) in liquid cultures and assessed *T. reesei* gene expression relative to each other and relative to glucose. Transcriptome data revealed that *T. reesei* up‐regulated many genes (over 100; necromass versus glucose substrate) coding for CAZymes, including enzymes that would target individual layers of an Ascomycota fungal cell wall. We also observed differential expression of protease‐ and laccase‐encoding genes on high versus low melanin necromass, highlighting a subset of genes (fewer than 15) possibly linked to the deconstruction of melanin, a cell wall constituent that limits necromass decay rates in nature. Collectively, these results advance our understanding of the genomic traits underpinning the rates and fates of carbon turnover in an understudied pool of Earth's belowground carbon, fungal necromass.

## Graphical abstract

We used RNA sequencing to characterise *Trichoderma reesei* RUT‐C30 genes and pathways involved in the decomposition of *Hyaloscypha bicolor* necromass (dead fungal cells and tissues). We showed that *T. reesei* up‐regulated genes involved in the utilisation of fungal cell wall components such as chitin, glucans, and mannoproteins. We also found that high melanin necromass possibly induced a transcriptional response that involved the up‐regulation of protease‐ and laccase‐encoding genes compared to low melanin fungal necromass.

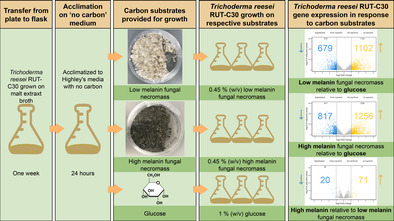

## 1 Introduction

A significant portion of soil carbon (C) on Earth is present in the form of soil organic matter (Pan et al. 2011; Bradshaw and Warkentin 2015). Although plant litter inputs were long considered to be the primary determinant of soil C stocks (Waksman 1936; Swift et al. 1979; Berg and McClaugherty 2008), there is growing recognition from soils under a variety of vegetation types that a significant fraction of the most persistent C bound in mineral associated organic matter (MAOM C), is microbial in origin (Clemmensen et al. 2013; Kallenbach, Frey, and Grandy 2016; Beidler et al. 2020). Both fungi and bacteria contribute to soil C stocks with their necromasses (dead cells and tissues), and fungal necromass has been shown 2–3 times higher than bacterial necromass in MAOM C (Liang et al. 2019; Lavallee, Soong, and Cotrufo 2020; Angst et al. 2021; Wang et al. 2021; Sokol et al. 2022).

Fungal necromass, which contains C and nitrogen (N) in both simple (sugars and amino acids) and polymeric forms (chitin, glucan, mannan and mannoprotein), is recycled by soil microbes via a complex, coexisting process involving biomass deposition and subsequent degradation (Starke et al. 2020; Lindahl et al. 2021; Jörgensen et al. 2022). The most abundant core components of fungal cell walls are β‐glucans and α‐glucans which are rich in carbon and can account for 50%–60% of the cell wall mass (Bartnicki‐Garcia 1968; Ryan et al. 2020). A key but variable component of fungal cell walls that also delineates fungi from plants is chitin (0.5%–20%; (Gaderer, Seidl‐Seiboth, and Kappel 2017)), which often forms complexes with β‐glucan (Drigo et al. 2012; Ryan et al. 2020; Chakraborty et al. 2021; Fernando et al. 2023). In the fungal phylum Ascomycota, fungal cell walls have an outer mobile surface shell that is made up of a combination of galactomannan (GM) and galactosaminogalactan (GAG), which can act to shield the rigid core of the glucan‐chitin complex (Chakraborty et al. 2021; Fernando et al. 2023). The mobile fraction of fungal cell walls among Ascomycota (e.g., *Aspergillus*) also harbour proteins (e.g., mannoproteins) and lipid components (Chakraborty et al. 2021; Fernando et al. 2023), and it is estimated that cell wall proteins harbour approximately 70% of fungal cell wall N (Smiderle et al. 2012).

Melanin is frequently an additional component of fungal cell walls, particularly for fungi in the Ascomycota (Butler and Day 1998; Butler and Day 1998; van der Wal et al. 2009). Melanin is a biopolymer composed primarily of phenolic and indolic monomers (Bull 1970; Ryan et al. 2020) that assists fungi in dealing with various forms of stress (Koide, Fernandez, and Malcolm 2014; Cordero and Casadevall 2017). Its concentration varies with tissue type and environment, and its presence hinders necromass decomposition similar to how lignin hinders plant litter decomposition (Fernandez and Koide 2014; Fernandez and Kennedy 2018; Maillard et al. 2023). Multi‐year necromass decomposition experiments have revealed that fungi and bacteria readily colonise and degrade fungal necromass (Maillard et al. 2021) and that there is a negative correlation between the presence of melanin in necromass and the decomposition of the necromass (Fernandez and Koide 2014; Fernandez and Kennedy 2018; Maillard et al. 2023). Factoring melanin alongside the other components in cell walls, the biochemical quality of necromass controls the rate and fate of its constituents during decay (Brabcová, Štursová, and Baldrian 2018; Beidler et al. 2020; Maillard et al. 2020) and promises some reliability to modellers attempting to make predictive trait‐function relationships.

In forests, necromass decomposition is driven by taxonomically and functionally rich fungal and bacterial communities that associate with necromass over both short and long periods (Kennedy and Maillard 2023). While mass loss can occur rapidly within days to weeks following necromass incubation in ground contact (Ryan et al. 2020), the studies of Maillard et al. (2021, 2023) documented diverse microbial communities associated with decomposing necromass over months and years, highlighting their potential to extract C and nutrients for energy throughout the necromass decomposition process. A number of bacterial and fungal guilds are present from early to late necromass decomposition, including saprotrophs, ectomycorrhizal fungi, mycoparasites and animal parasites (Brabcová et al. 2016; Brabcová, Štursová, and Baldrian 2018; Maillard et al. 2020). A core necrobiome was defined recently by Cantoran et al. (2023), with members of the Ascomycota genus *Trichoderma* being one of the most common (10, of 18 fungal genera) on necromass across different forest biomes, particularly abundant in early‐ and mid‐decay stages. *Trichoderma* is ecologically diverse, consisting of both saprotrophic and mycoparasitic species (Chaverri and Samuels 2013), and the extensive development of some isolates for biotechnological purposes makes it a tractable subject for genomic analyses in decaying necromass.

Given this relevance of *Trichoderma*, we selected the model fungus *Trichoderma reesei* (strain RUT‐C30), which carries a truncated *cre1* gene allowing glucose derepression (Ilmén, Thrane, and Penttilä 1996), to understand its gene expression patterns in the deconstruction of necromass in relation to glucose without the confounding effect of catabolite repression. The genome of *T. reesei* also has an expanded repertoire of GH18 chitinase‐encoding genes, which has been linked to a lifestyle of eating fungal biomass as a mycoparasite (Martinez et al. 2008). Necromass substrates included a low and high melanin phenotype, both derived from the Ascomycete fungus *Hyaloscypha bicolor* in different culturing conditions (Fernandez and Kennedy 2018). We conducted growth experiments in liquid media, we extracted RNA, and we quantified the transcriptional responses of *T. reesei* to the different carbon substrates using RNA‐seq. We found that *T. reesei* responded differentially to the tested carbon substrates, with enzymes specific to chitin, glucan and mannan being up‐regulated by *T. reesei* on necromass substrates compared to glucose. Furthermore, we observed that proteases and laccases, both potentially involved in melanin degradation, were up‐regulated on the higher melanin version of the *H. bicolor* necromass. Collectively, our study showed that *T. reesei* could readily utilise C and N embedded in fungal cell walls, and it implicated several gene regulation pathways in controlling, and thus limiting the rate of the release of C and N in decaying necromass.

## 2 Results

### 2.1 *T. reesei* Expressed Genes Differently on Necromass Than on Glucose

**FIGURE 1:**
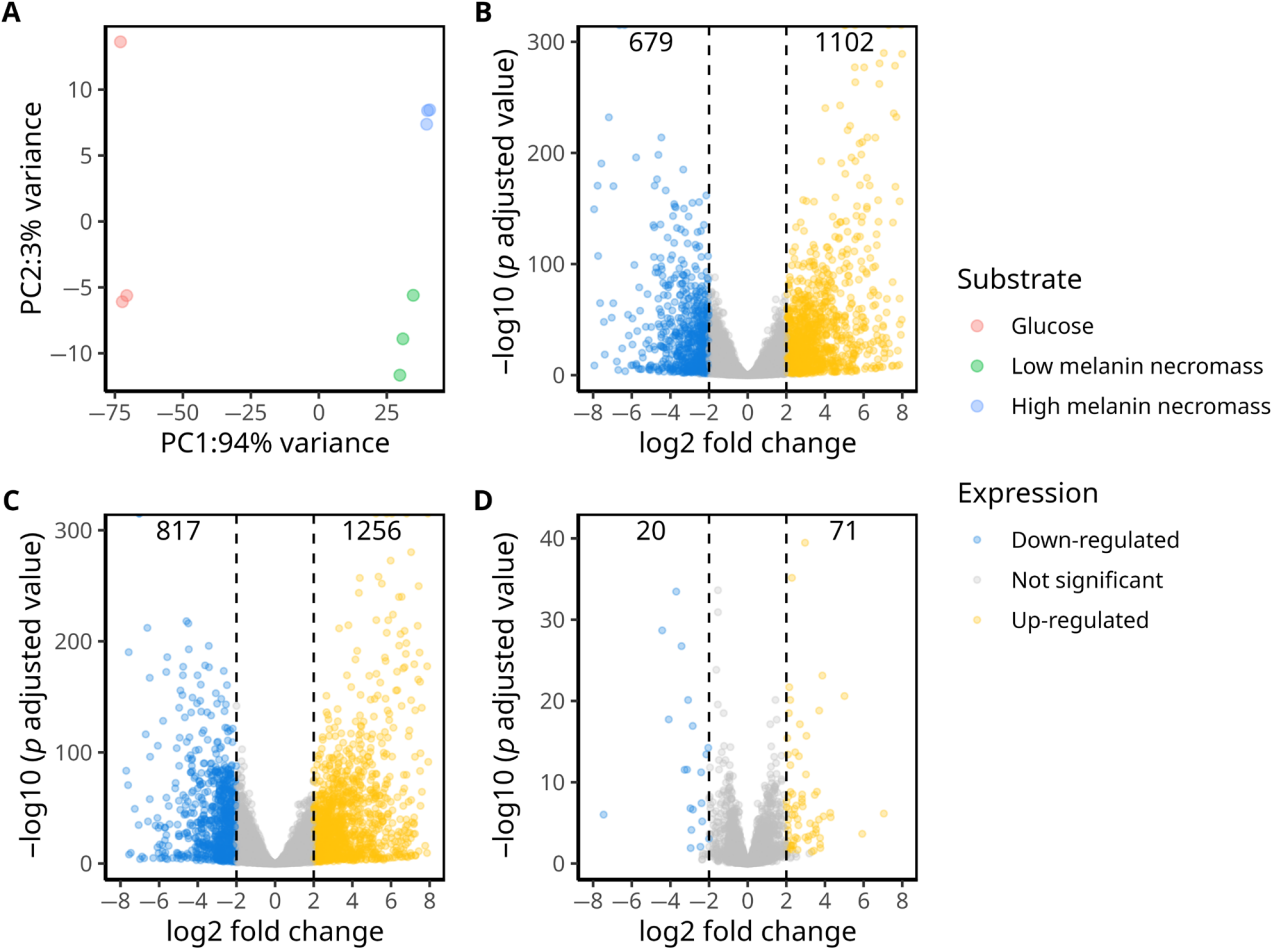
*Trichoderma reesei* responded differentially to melanized necromass. (A) Principal component analysis revealed variation in gene expression profiles of *T. reesei* growing on melanized fungal necromass and glucose. In (B), the number of differentially expressed genes between low melanin fungal necromass and glucose treatment are shown. The number of differentially expressed genes between high melanin fungal necromass treatment and glucose are shown in (C), whereas in (D) the number of genes differentially expressed between high melanin and low melanin fungal necromass treatments are shown.

Using a strain of *T. reesei* lacking glucose catabolite repression, we determined the whole transcriptome response of *T. reesei* to three substrates (glucose, low melanin necromass from *H. bicolor* and high melanin necromass from *H. bicolor* ). Principal components analyses based on RNA‐seq gene expression profiles of samples from the three treatments revealed a clear separation of all samples according to substrate type. Expression profiles of *T. reesei* growing on low and high melanin necromass, expectedly, showed large variation from those growing on glucose (Figure 1A; PC1). The expression profiles of *T. reesei* growing on low melanin necromass were also different (Figure 1A; PC2; average = −8.72) from those growing in the presence of high melanin necromass (Figure 1A; PC2; average = 8.08), distinguishable in ordination space (Figure 1A; PC2). We performed an analysis of differentially expressed genes across the three treatments using a cutoff threshold of log2 fold change ≥ 2 and ≤ −2; *p* (adjusted) < 0.05 (Table S1). This analysis revealed a differential response of *T. reesei* to both types of necromass as compared to glucose (Figure 1B–D).

### 2.2 Transcriptional Response to Necromass Was Typically Up‐Regulation, Including CAZyme Genes

**FIGURE 2:**
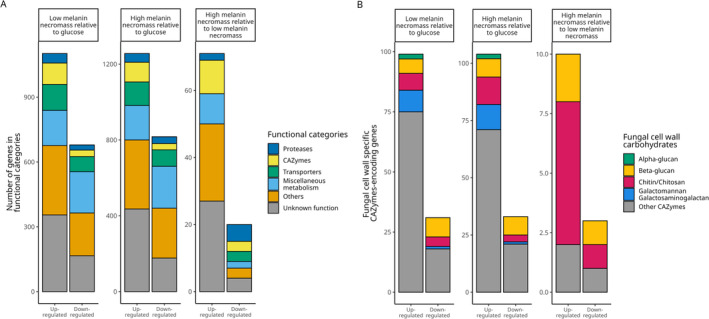
Distribution of differentially expressed genes into broad functional categories across treatments. (A) All differentially expressed genes with the threshold of log2 fold change ≥ 2 and ≤ –2 (*p* adjusted < 0.05) are organised into six categories, with CAZymes as a category of interest in the decomposition of necromass. While ‘Others’ include genes that do not fall into the four categories (CAZymes, Proteases, Transporters and Miscellaneous metabolism) based on their annotations, ‘unknown functions’ category includes genes that have no annotation as per the Joint Genome Institute (JGI) database hosted at https://mycocosm.jgi.doe.gov/mycocosm/home. (B) All differentially expressed CAZymes‐encoding genes are categorised based on their carbohydrate targets in the fungal cell wall, indicating a role in the decomposition of fungal cell wall components (e.g. α‐glucans, β‐glucans, chitin, galactomannan and galactoseaminogalactan).

To ascertain how *T. reesei* responds to low and high melanin necromass from *H. bicolor* in comparison to glucose, we binned differentially expressed genes into six major functional categories (Figure 2A; Zhang et al. 2016; Anderson et al. 2022). This enabled three pairs of substrate comparisons among the six gene categories. It is important to note two things: First, by using the RUT‐C30 strain of *T. reesei*, we could eliminate glucose repression of CAZyme genes and focus comparisons on complex carbon catabolism. Second, we knew from our PCA analyses that the low versus high melanin comparisons would reveal a smaller, more targeted set of differentially expressed genes that we could assume were either related to melanin deconstruction or a starvation response. We could thus answer what genes are involved in necromass deconstruction, broadly, as well as answer what genes are activated by the presence of the rate‐limiting constituent in the substrate, melanin.

When growing on low melanin necromass, 355 up‐regulated genes were attributed to ‘unknown function’, relative to glucose, while only 166 unknown function genes were down‐regulated (2.1× differential). Similarly, there was a 1.6× differential for up‐regulated versus down‐regulated genes in the ‘others’ category (321 up; 198 down), a 1.7× differential in ‘membrane transporters’ (120 up; 70 down), and a 1.8× differential in ‘proteases’ (44 up; 24 down). Genes in the ‘miscellaneous metabolism’ category had 1.2× more down‐regulated genes (163 up; 191 down). The highest differential (2.3×) observed was for genes encoding CAZymes, with 99 up‐regulated genes compared to 30 down‐regulated genes.

The differential gene expression patterns from the high melanin necromass versus glucose were similar to those of low melanin necromass versus glucose. More genes were up‐regulated for ‘unknown function’ genes (436 up; 177 down), for ‘others’ genes (364 up; 263 down), for transporter‐encoding genes (124 up; 87 down), and for proteases (46 up; 36 down). Differentially expressed genes in the ‘miscellaneous metabolism’ category included 182 up‐regulated and 221 down‐regulated genes. Similar to the low melanin necromass versus glucose, there was 3.2× more up‐ than down‐regulated CAZyme‐encoding genes (104 up; 33 down).

Comparing expression between high and low melanin necromass, only 91 genes were differentially expressed and only in a portion of our gene function categories (Figure 1D). Genes in the ‘unknown function’ category included more up‐regulated than down‐regulated genes (27 up; 4 down) (Figure 2A). There were more up‐regulated genes in the ‘others’ category, as well (23 up; 3 down). Unlike other treatment comparisons, fewer genes encoding proteases were up‐regulated compared with down‐regulated (2 up; 5 down). The ‘CAZymes’ category genes were again more up‐ than down‐regulated on high melanin necromass (10 up; 3 down).

### 2.3 CAZyme Gene Families Specific to Fungal Cell Wall Components Were Up‐Regulated on Necromass

To add clarity relative to melanin and other necromass‐specific decay pathways within the CAZymes, we further divided CAZymes based on the annotation and functional assignments of genes into six broadly defined categories (Figure 2B). In this mannose‐focused section of the article and the following sections of our Results, we detail these categories by fungal cell wall component—mannan/mannoproteins on the outermost layer, α‐ and β‐glucan, chitin/chitosan and finally melanin‐linked genes toward the base layer near the fungal cell membrane.

#### 2.3.1 Mannose and Mannoprotein Components

**FIGURE 3:**
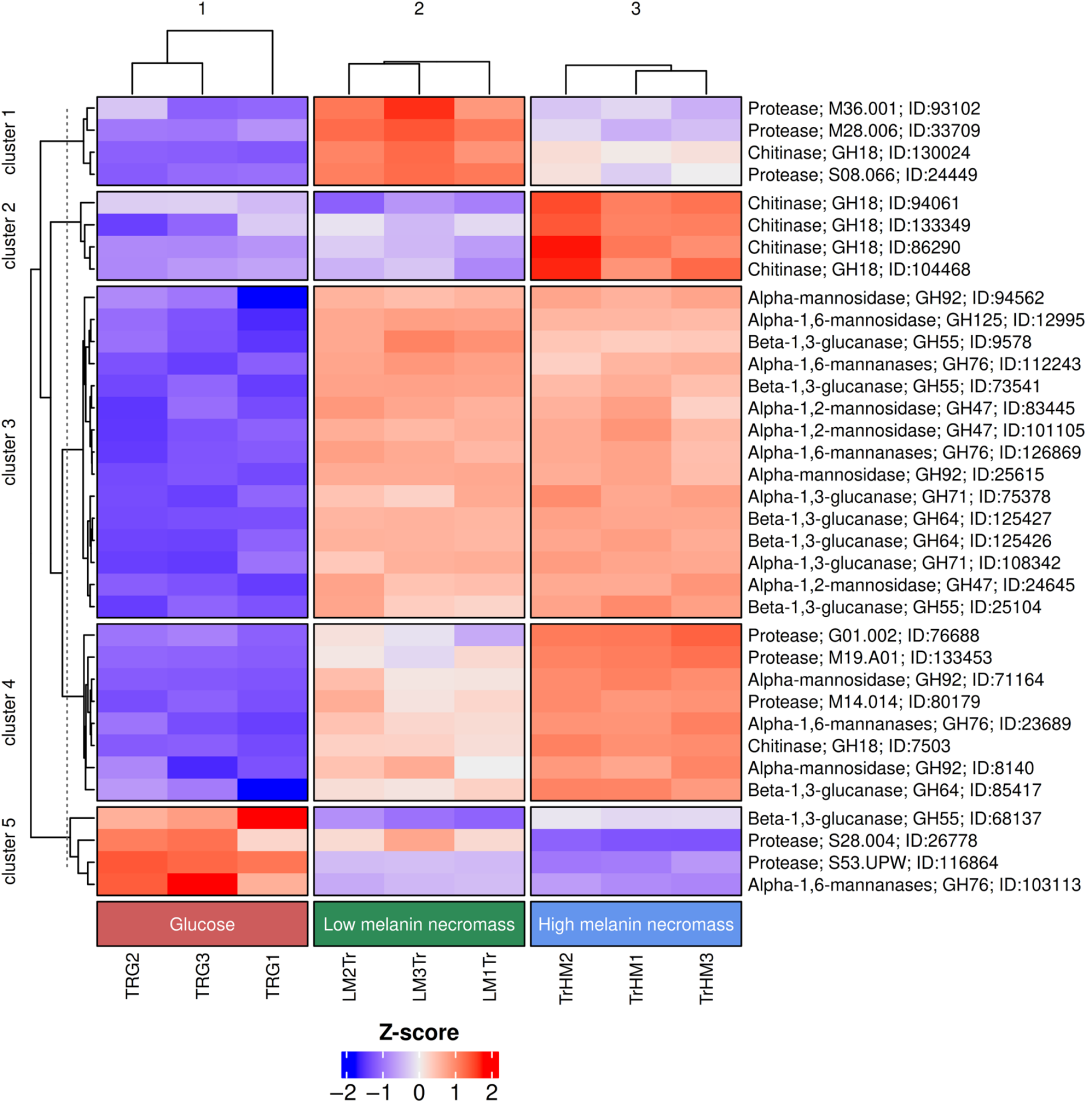
Heatmap of selected genes with significant differential expression (Benjamini–Hochberg adjusted *p* < 0.05) between glucose (1% w/v), low melanin fungal necromass (0.45% w/v) and high melanin fungal necromass (0.45% w/v) treatments. Differentially expressed genes were grouped into five clusters based on their expression across the three treatments, represented by the *Z*‐score (row scaling) of each gene. Each treatment is represented by three independent biological replicates. For maintaining the default order of all treatments in this figure, that is, glucose (red), low melanin necromass (green) and high melanin necromass (blue), ‘cluster_column_slices’ in ComplexHeatmap was set as ‘FALSE’.

The cell walls of many Ascomycota fungi contain galactomannan (GM) and galactosaminogalactan (GAG) presented on the outermost layer, with GM containing α‐1,2‐mannose, α‐1,6‐mannose and galactofuranose sugar units, while the GAG is made up of N‐acetylgalactosamine, galactoseamine and galactopyranose (Chakraborty et al. 2021; Fernando et al. 2023). Compared to our glucose treatment, both the low melanin and high melanin necromass treatments resulted in up‐regulation of mannan‐ and mannoprotein‐active CAZyme genes (Figure 2B). Genes encoding a diverse set of CAZyme families (GH47, GH76, GH92 and GH125) implicated in the removal of mannose from GM and GAG were overexpressed on necromass as compared to glucose (Figures 3 and S1). Among these genes, three GH47‐encoding (mannosyl‐oligosaccharide α‐1,2‐mannosidase) genes were up‐regulated on both necromass treatments, as compared to glucose (cluster 3 in Figure 3). Similarly, three GH76‐encoding (endo‐α‐1,6‐mannanase) genes, four GH92‐encoding (exo‐acting α‐mannosidase) genes, and one GH125‐encoding (exo‐α‐1,6‐mannosidase) gene were up‐regulated by *T. reesei* when it was grown on either necromass type, compared to glucose (clusters 3 and 4 in Figure 3). Only one GH76‐encoding gene was down‐regulated on necromass (cluster 5 in Figure 3).

#### 2.3.2 Glucan Components

Glucans (α‐ and β‐linked) are the most abundant components of most fungal cell walls, and they maintain cell wall strength and rigidity. The genome of *T. reesei* contains several genes that encode glucanases, and these belong to different CAZyme families. Although a major biotechnological reason to study this fungus has been their high titre production of β‐1,4‐glucanases (cellulases, particularly exo‐acting), there are several CAZyme families more relevant for this study that act on the prevalent β‐1,3‐glucan in fungal cell walls (Santos et al. 2020), including GH16, GH17, GH55, GH64 and GH128. CAZyme families active on β‐glucan were comparable across our treatments (Figure 2B), but two of these families, GH55 and GH64, showed a consistent response to necromass, as compared to glucose (cluster 3 and 4 in Figure 3). Three GH55 family genes encoding β‐1,3‐glucanases were differentially expressed (2 up; 1 down; log2 fold change ≥ 2 and ≤ −2; *p* (adjusted) < 0.05) on necromass, compared to glucose (cluster 3 and 5 in Figures 3 and S2A). Three GH64 family genes were up‐regulated on high melanin necromass, and two GH64 genes were up‐regulated on low melanin necromass (Figure S2B), in comparison to their expression on glucose.

The differentially expressed genes encoding other glucan‐active CAZyme families were more variable. Three genes encoding GH17 and two genes encoding GH128 family CAZymes were down‐regulated in the presence of necromass as compared to glucose (Figure S3). When we compared the low melanin necromass treatment with glucose, two GH16‐encoding genes were up‐regulated (protein ID = 103726 and 93445) and two genes were down‐regulated (Figure S4). A total of five genes encoding GH16 family of glucanases were differentially expressed when we compared high melanin necromass treatment with glucose. Of these, three genes were up‐regulated (protein ID = 103726; 93445; [protein ID = 95463; log2 fold change = 1.8]) in the presence of high melanin necromass whereas two genes were down‐regulated (Figure S4). Comparing the low and high melanin treatments revealed two genes (protein ID = 93445 and 95463) up‐regulated and one gene (protein ID = 111701) down‐regulated in the presence of high melanin necromass, although the same gene was also up‐regulated on glucose (Figure S4). There was a single GH30_3‐encoding gene (active on β‐1,6‐glucan) that was down‐regulated in the presence of both low and high melanin necromass, as compared to glucose (Figure S4).

In addition to β‐glucan, the cell walls of fungi in the Ascomycota, such as those of *Aspergillus* species, also contain α‐glucan (Chakraborty et al. 2021; Fernando et al. 2023). The α‐glucan‐active CAZyme families that were differentially expressed were all up‐regulated on necromass (Figure 2B), with *T. reesei* up‐regulating two genes (*p* (adjusted) < 0.05) that encode α‐1,3‐glucanases in the GH71 family (cluster 3 in Figures 3 and S2C).

#### 2.3.3 Chitin and Chitosan Components

A hallmark of many fungi is the presence of chitin in the cell wall, providing tensile strength and rigidity by forming complexes with β‐glucan within the cell wall matrix. A key difference between treatments with low and high melanin necromass was the up‐regulation of chitin‐active CAZyme genes when growing on high melanin necromass (Figure 2B). Six genes encoding chitinase enzyme family GH18 were differentially expressed when *T. reesei* was grown in the presence of high melanin necromass compared to low melanin necromass (cluster 1, 2 and 4 in Figures 3 and S5). Of these, five genes were up‐regulated (log2 fold change ≥ 2; *p* (adjusted) < 0.05) in the presence of high melanin necromass (cluster 2 and 4 in Figure 3), whereas one gene was down‐regulated (log2 fold change ≤ −2; *p* (adjusted) < 0.05; cluster 1 in Figure 3). Regarding chitin degradation, we also identified that a gene (protein ID = 135815) encoding lytic polysaccharide monooxygenase (LPMO) belonging to the auxiliary activity (AA) family AA11 was up‐regulated by *T. reesei* in response to high melanin necromass as compared with low melanin necromass (Figure S6). While two other AA11‐encoding genes did not meet the cutoff criteria, we noticed that one of the genes (protein ID = 97768) was up‐regulated (log2 fold change = 1.2) in the presence of both low and high melanin necromass as compared to glucose. The other gene (protein ID = 71162) was down‐regulated in the presence of high melanin necromass compared to both glucose and low melanin necromass (Figure S6).

In addition to GH18, CAZyme families active on chitosan and/or chitin were differentially expressed, including GH75 and GH89 when comparing necromass to glucose treatments. We found that a chitosanase‐encoding gene (protein ID = 57859; GH75), an N‐acetylglucosaminidase‐encoding gene (protein ID = 71653; GH89), and a GH18 encoding gene (protein ID = 142123) were up‐regulated (log2 fold change ≥ 2; *p* (adjusted) < 0.05) in the presence of glucose, compared to necromass (Figure S7). Overall, however, most chitinase‐encoding genes in *T. reesei* were up‐regulated on necromass, compared to glucose.

#### 2.3.4 Melanin and Protein Components

As melanin is not a single chemical compound, it is a challenge to quantify (colour can qualify its presence/abundance), and it may be present in the outermost, innermost or middle layers of fungal cell walls, or be uniformly integrated (Eisenman and Casadevall 2012). Some relevant proteases were differentially expressed when *T. reesei* was grown on *H. bicolor* necromass, compared to glucose, and two protease‐encoding genes were up‐regulated on high versus low melanin necromass (log2 fold change ≥ 2; *p* (adjusted) < 0.05), including a metalloprotease gene (protein ID = 133453; M19) and a glutamic protease gene (protein ID = 76688; G1) (cluster 4 in Figures 3 and S8). Although expression of a gene (protein ID = 80179) encoding a carboxypeptidase MeCPA (family M14) did not meet the fold change threshold (log2 fold change ≥ 2), it was notably up‐regulated (log2 fold change = 1.1; *p* (adjusted) < 0.05) when *T. reesei* was grown on high melanin necromass (cluster 4 in Figures 3 and S8). We also found that five genes were down‐regulated on high melanin necromass in comparison to low melanin necromass (log2 fold change ≤ −2; *p* (adjusted) < 0.05). These included two genes encoding for metalloproteases (protein ID = 33709; M28) (protein ID = 93102; M36) (cluster 1 in Figures 3 and S8) and three genes encoding for serine proteases (protein ID = 24449; S08) (protein ID = 26778; S28) (protein ID = 116864; S53) (cluster 1 and 5 in Figure 3). However, genes encoding serine protease family S28 and S53 were also up‐regulated in the presence of glucose (log2 fold change ≥ 2; *p* (adjusted) < 0.05; cluster 5 in Figures 3 and S8).

Interestingly, *T. reesei* also differentially expressed three laccase‐encoding genes (AA1; Figure S9) when on high melanin necromass. Of these laccase genes, which have often been linked to lignin degradation/modification in other saprotrophic fungi, two were up‐regulated (protein ID = 104519 [log2 fold change > 2] and 88862[log2 fold change = 1.2]), and one was down‐regulated (protein ID = 92940; log2 fold change = −0.92) on high versus low melanin necromass.

## 3 Discussion

In this study, we examined the gene expression of a model decomposer on fungal necromass relative to glucose as a positive control, using the Ascomycota *T. reesei* RUT‐C30 strain to avoid repression of complex carbohydrate gene expression. We also included a comparison of low versus high melanin necromass, given that many studies have indicated that melanin limits necromass turnover rates in nature (Fernandez et al. 2016). Because *Trichoderma* spp. have been consistently observed to abundantly associate with decaying necromass in the field, particularly in the early‐ and mid‐decay stages (Cantoran et al. 2023; Maillard et al. 2023), and because of the long history of *T. reesei* as a genomics model system, we used *T. reesei* as a decomposer to characterise genes involved in necromass decomposition.

We found that *T. reesei* overexpressed genes responsible for the utilisation of the largest fungal cell wall fractions (galactomannans; α‐ and β‐glucans) in both low and high melanin necromass compared to glucose. Galactomannan and glucans, respectively, comprise the less dense outer shell and the more rigid core of the cell walls of fungi in the Asocmycota, such as *H. bicolor* (Kang et al. 2018; Chakraborty et al. 2021; Fernando et al. 2023). The up‐regulation of genes encoding α‐1,2‐mannosidase and α‐1,6‐mannosidase might indicate the ability of *T. reesei* to target and remove mannose in the outer layer galactomannan. *Trichoderma* are common fungal biocontrol agents, and as a relevant example, the species *T. harzianum* has been shown to overexpress mannosidase‐encoding genes when encountering phytopathogenic fungi such as *Fusarium solani*, *F. oxysporum* and *Scleortinia sclerotiorum* (Ramada et al. 2016; Nauom et al. 2019).

Once accessing glucans in necromass, *T. reesei* may be induced to express GH71 α‐1,3‐glucanases, known from plant pathogenic *Trichoderma* species to target cell wall α‐glucans (Ait‐Lahsen et al. 2001; Ramada et al. 2016). While several CAZyme families are involved in the degradation of β‐1,3‐glucans (Santos et al. 2020), our data showed that compared to glucose, GH55, GH64 and three GH16‐encoding genes were up‐regulated in response to necromass. Previous research has shown that *Trichoderma* species can overexpress its β‐1,3‐glucanase‐encoding genes (belonging to different CAZyme families) when fungal cell wall components are provided as a carbon substrate (Ramada et al. 2016; Nauom et al. 2019). The only contrast to the above patterns were GH128‐, GH17‐ and two GH16‐encoding genes that were up‐regulated on glucose in comparison to growth on necromass, a pattern duplicated in chitosanase (GH75) and N‐acetylglucosaminidase (GH89). These patterns on glucose treatments could collectively indicate *T. reesei* cell wall remodelling (Gruber and Seidl‐Seiboth 2012) at a later growth stage on an exhausted glucose substrate (White et al. 2002), possibly to relieve stress (Pitson, Seviour, and McDougall 1993; Ramada et al. 2016; Gaderer, Seidl‐Seiboth, and Kappel 2017) and to capture components (e.g., N‐acetylglucosamine) released back into the liquid culture medium (Perez‐Leblic et al. 1982).

In accessing chitin, which we assume is closer to the necromass fungal cell membrane (Kang et al. 2018; Chakraborty et al. 2021; Fernando et al. 2023), *T. reesei* overexpressed familiar chitinase‐encoding genes. Chitinases have been shown to up‐regulate in response to exogenous as well as embedded fungal cell wall chitin among a wide range of filamentous fungi, including several species of *Trichoderma* (Viterbo et al. 2001; Samolski et al. 2009; Gruber and Seidl‐Seiboth 2012; Junges et al. 2014). The pattern of up‐regulation of chitinase‐encoding genes (GH18 family) on necromass, with a higher expression on high melanin necromass, suggests that *T. reesei* might have to invest more energy to access chitin shielded by melanin in the cell wall matrix (Bull 1970). The up‐regulation of AA11‐encoding genes (lytic chitin monooxygenase) on higher than low melanin necromass might be for the same reason.

Regarding melanin, it is harder to pinpoint candidate genes, but the proteases and laccase up‐regulated on high melanin necromass were notable. Proteases have been implicated in the degradation by *Metarhizium* spp. of melanin in insect exoskeletons (St. Leger and Wang 2020), using proteases to breach the outer cuticle in a living insect (Joshi and St. Leger 1999; Freimoser et al. 2003) by inactivating the insect host's prophenoloxidases (Huang et al. 2020). Among biocontrol‐relevant *Trichoderma* species, protease‐encoding genes are often up‐regulated in response to phytopathogenic fungal cell wall components (Steindorff et al. 2014; Ramada et al. 2016; Nauom et al. 2019). For example, during combative interactions with *Verticillium dahliae* on agar plates, *Trichoderma atroviride* overexpressed its metallocarboxypeptidase (M14)‐encoding gene to perhaps target the cell wall structure of its opponent (Morán‐Diez et al. 2019). The involvement of laccase in the degradation of melanin is widespread across fungi with different ecological lifestyles. Catalano et al. (2011) showed that the mycoparasitic *T. virens* can degrade melanized sclerotia of *Botrytis cinerea* and *S. sclerotiorum* using a laccase. Similarly, *Phlebia radiata* and *Phanerochaete chrysosporium*, white rot fungi, use laccase to decolorize melanin from *Cladosporium* spp., (Kaneko et al. 2009) and to degrade sclerotial melanin from *S. sclerotiorum* (Butler, Gardiner, and Day 2009), respectively. Entomopathogenic fungi also express laccase in appressoria to directly disrupt cuticular melanin production (Fang et al. 2010), and in a recent study laccase from *Beauveria bassiana* was shown to interfere with the host phenoloxidase activation, suggesting a role in the oxidation of phenol substrates that the insect used to synthesise melanin (Lu et al. 2021). If melanin degradation is the key to predicting rates of necromass turnover, our results provide protease and laccase candidates to target future work.

Despite demonstrating key transcriptional responses of *T. reesei* in the degradation of *H. bicolor* necromass, we acknowledge that our study has limitations. First, our data is based on liquid‐grown cultures, which are different from a natural environment such as soil. Ideally, a solid‐state design would have been closer to a soil setting, but in our pilot tests, we were unable to get quality RNA (RINs < 4, in many cases) during destructive sampling and RNA extractions. We had this issue in two, full‐scale, separate attempts, which we believe was due to inhibition from unknown necromass components. We note this as a caution for anyone attempting ‘litter bag’ transcriptomics, including meta‐transcriptomics in field trials. Second, the lack of temporal expression data limits any ability to identify genes that might have an expression pattern dependent on time, such as described in the degradation of wood for brown rot fungi (Zhang et al. 2016; Anderson et al. 2022). Particularly for the glucose treatment, our use of RUT‐C30 eliminates the repressive effect of glucose on complex polysaccharide enzyme‐encoding genes, but it does not affect transcription patterns for all CAZyme‐ or protease‐encoding genes, and it could speed cellular respiration and result in comparisons at different growth phases.

Overall, the results we obtained provide an essential foundation for mechanistic studies of how fungi deconstruct necromass, an important yet understudied soil ‘litter’ fraction for C and N cycling that must be addressed (Wang et al. 2021; Buckeridge, Creamer, and Whitaker 2022). In particular, we showed that enzymes active on chitin, glucan, and mannose were overexpressed by *T. reesei* when grown on necromass, which underscores the potential for fungi, along with other microbes (Starke et al. 2020), to utilise C‐ and N‐containing compounds in fungal cell walls. Our study also spotlights a specific set of laccases and proteases that may play a key role in the removal of melanin embedded in the necromass. Determining the extent to which other fungi and bacteria utilise similar or different genes to degrade fungal necromass, particularly with differing levels of melanization, represents a key next step in further understanding how microbial‐derived C and N are cycled and retained in soils.

## 4 Materials and Methods

### 4.1 Fungal Strains and Growth Conditions

*T. reesei* was routinely maintained on malt extract agar in Petri plates. Aseptically, two plugs from the agar plates of *T. reesei* were introduced to malt extract broth medium and flasks were incubated at 26°C and 100 rpm on an Innova 2300 platform shaker (New Brunswick Scientific, USA). After 1 week of incubation, *T. reesei* produced biomass that was harvested using a glass funnel (Fisherbrand, USA) with Whatman qualitative filter paper. The resulting fungal biomass was washed twice with autoclaved distilled water and chopped into pieces in a sterilised glass Petri dish with the help of a sterilised tweezer and knife. Next, we transferred the double‐washed biomass for acclimation to 500 mL Erlenmeyer's flasks (Pyrex, USA) containing 250 mL of Highley's media (Highley 1973; Zhang and Schilling 2017) that lacked any carbon substrate. Flasks were incubated for 24 h at 26°C and 100 rpm on the Innova 2300 platform shaker. After 24‐h acclimation, *T. reesei* biomass was transferred to 125 mL Erlenmeyer's flasks (Pyrex, USA) containing 50 mL Highley's media supplemented with either 1% (w/v) glucose or 0.45% (w/v) low or high melanin fungal necromass as carbon substrates. All flasks were incubated for 7 days at 26°C and shaken at 100 rpm on the Innova 2300 platform shaker.

### 4.2 Low Melanin and High Melanin Necromass From *H. bicolor*

*H. bicolor* (Hambl and Sigler) Vohník, Fehrer and Réblová (formerly *Meliniomyces bicolor*) cultures were grown on half‐strength potato dextrose (HiMedia Laboratories, PA, USA) agar (PDA) plates covered with a gel drying film (Promega, WI, USA) to avoid introducing agar plugs into liquid cultures. *H. bicolor* cultures were maintained in dark conditions at 23°C for 3 weeks. Next, mycelial plugs were transferred to liquid PD broth (PDB), with the pH adjusted to 5 using 10% HCl. Cultures were grown in 125 mL Erlenmeyer glass flasks filled with either 40 or 110 mL of liquid medium to generate low and high melanin biomass, respectively (Fernandez and Kennedy 2018). The cultures were incubated on orbital shakers run at 120 rpm for the low melanin and 150 rpm for the high melanin biomasses for 30 days at 25°C. After the incubation, all of either low or high‐melanin *H. bicolor* mycelium was harvested in bulk onto separate sterile sieves and rinsed with sterile deionised water (diH_2_O) to remove the remaining traces of the liquid medium. Next, the mycelium of each melanin type was homogenised using a mortar and pestle, transferred to sterile 50 mL centrifuge tubes (Fisherbrand, PA, USA), and stored at −80°C overnight. Tubes were then placed into a benchtop Freeze Dryer (Labconco, NH, USA) for 3 days at −50°C under vacuum to create the two necromass types used in this study.

We analysed the biomass of *H. bicolor* using Pyrolysis gas chromatography–mass spectrometry (Py‐GCMS), to determine major chemical components in low melanin and high melanin necromass (Ryan et al. 2020). Sugars and lipids were the two major constituents of both low melanin and high melanin necromass. The relative abundances of sugars in low melanin and high melanin necromass were 0.57 (std. = 0.027) and 0.44 (std. = 0.018), respectively. Moreover, the relative abundance of lipids in low melanin (0.29; std. = 0.023) and high melanin (0.27; std. = 0.006) necromass was comparable. The key difference between the necromass types was the N‐containing component, which was relatively more abundant in high melanin necromass (0.10; std. = 0.006) as compared to low melanin necromass (0.04; std. = 0.001). The relative abundance of the unspecified components in the two necromass types was higher in high (0.16; std. = 0.007) than low melanin necromass (0.08; std. = 0.008). The relative abundances of aromatic and sterols in both necromass types were significantly lower than the rest of the chemical components.

### 4.3 Total RNA Extraction and Purification

Total RNA was extracted from all samples on the same day, following the protocol described elsewhere (Zhang et al. 2016). Briefly, fungal biomass was harvested from the liquid culture with vacuum filtration using Büchner funnels and immediately snap‐frozen with liquid nitrogen. Once frozen, biomass was macerated with mortar and pestle and immediately transferred to pre‐chilled 1.5 mL microcentrifuge tubes. We added 1 mL TRIzol reagent to each sample and vortexed it for 15–30 s. Tubes were then incubated at room temperature for 2 min and afterwards put on ice. To remove cell debris, each sample was centrifuged at 10000 rpm for 10 min and approximately 1000 μL of supernatant was transferred to new 1.5 mL tubes. Next, 200 μL of chloroform was added to each sample and tubes were vigorously shaken with hands for 15 s. Samples were incubated at room temperature for 2 min and then centrifuged at 10000 rpm for 10 min at 4°C. Approximately 500 μL of the upper aqueous phase was transferred to new tubes, and an equal volume of 70% ethanol was added to each sample. PureLink RNA mini kit (Life Technologies, CA, USA) was used for binding, washing and purification of total RNA (Zhang et al. 2016). All samples were treated with DNase using the PureLink DNase kit (Life Technologies, CA, USA), which is compatible with the PureLink RNA mini kit to perform on‐column DNase treatment. RNA integrity and quality were checked using gel electrophoresis. High‐quality RNA with average RIN values exceeding 8 were used for library preparation. This RIN threshold of 8 was easily attainable in this liquid culture set‐up (RINs > 9, commonly), but notably was unattainable (RINs < 4, commonly) in multiple pilot study attempts using necromass presented in litter bags on semi‐solid agar media. This challenge is discussed in our Discussion section, and we assert should be a consideration for others in their future work, including field meta‐transcriptomics.

### 4.4 Library Preparation, RNA Sequencing and Data Analyses

Total RNA samples were submitted to Genewiz (Azenta Life Sciences; New Jersey) for cDNA library preparation with the NEBNext Ultra II RNA Library Prep kit. Poly(A) tail capturing technique was used to harvest only mature mRNA from the total RNA population, thereby leaving rRNA behind. All samples were sequenced using the Illumina HiSeq 4000 platform (Illumina, San Diego, CA) with 2 × 150 bp paired‐end configuration and chemistry.

### 4.5 RNA‐Seq Data Analyses

Raw data were processed with Collection of Hierarchical UMII‐RIS Pipelines (CHURP; v0.2.2), developed by the Supercomputing Institute of the University of Minnesota (Baller et al. 2019), and the trimmed and quality‐controlled reads were used in downstream analyses. Kallisto v0.48.0 (Bray et al. 2016) was used for building the index file and the quantification of transcripts using *T. reesei* RUT‐C30 reference transcriptome (cDNA sequences) downloaded from Ensembl Fungi (https://fungi.ensembl.org/Trichoderma_reesei_rut_c_30_gca_000513815/Info/Index) on April 19, 2023. Transcript abundances from kallisto were imported into R Statistical Software (v4.3.2; R Core Team, 2023) via the taximport v1.30.0 package (Soneson, Love, and Robinson 2015) and analysed using the DESeq2 v1.42.0 (Love, Huber, and Anders 2014) to determine differentially expressed genes between the treatments. Prior to running the DESeq2 functions, we performed pre‐filtered low‐count genes from our dataset using the script: keep ← rowSums (counts (dds) > = 10) > = 3. To normalise samples by the sequencing depth of their respective libraries, we implemented standard DESeq2 functions with default parameters. The Wald test was used to determine statistical significance using the negative binomial generalised linear model. *p* values correction for multiple testing was performed with the Benjamini–Hochberg procedure. In order to generate a heatmap, we transformed the regularised log count data (Love, Huber, and Anders 2014) to the Z‐scale, using scaling per row. Heatmap was generated in R using the ComplexHeatmap package (Gu, Eils, and Schlesner 2016; Gu 2022). Finally, ggplot2 v3.4.4 (Wickham 2016) was used to visualise the analysed data.

## Author Contributions

**Irshad Ul Haq:** methodology, investigation, writing – original draft, writing – review and editing, visualization, software, formal analysis, data curation, validation. **Peter Kennedy:** conceptualization, funding acquisition, writing – review and editing, methodology, supervision, resources, project administration, investigation. **Kathryn M. Schreiner:** writing – review and editing, supervision, resources, methodology. **Julia C. Agnich:** methodology, writing – review and editing, data curation. **Jonathan S. Schilling:** supervision, resources, project administration, conceptualization, funding acquisition, writing – review and editing, investigation.

## Conflicts of Interest

The authors declare no conflicts of interest.

## Supporting information


**Table S1.** List of differentially expressed genes (DEGs) with a cutoff threshold of log2 fold change ≥ 2 and ≤ −2; *p* (adjusted) < 0.05, across three treatments.

**Figure S1.** Supplementary Figures.

## Data Availability

Data are available at NCBI under the BioProject number PRJNA1097855 and the Gene Expression Omnibus (GEO) database under the GEO submission number GSE263516. All codes and scripts used in the analysis of data presented in this study are publicly available at https://github.com/IrshadUlHaq1/necromass.
