# Supplementary material for: Gene Expression by a Model Fungus in the Ascomycota Provides Insight Into the Decay of Fungal Necromass
Source: Environ Microbiol. 2024 Dec 8;26(12):e70006. doi: 10.1111/1462-2920.70006 (PMC11625536; doi:10.1111/1462-2920.70006)

Supplementary Data (Supplementary Figures 1-9)

Gene expression by a model fungus in the Ascomycota provides insight into the decay of fungal necromass

Irshad Ul Haq[1], Peter Kennedy[1], Katie Schreiner[2,3], Julia C. Agnich[3] Jonathan S. Schilling[1#]

[1]Department of Plant and Microbial Biology, College of Biological Sciences, University of Minnesota, Twin Cities
[2]Department of Chemistry and Biochemistry, University of Minnesota Duluth, 1038 University Dr., Duluth, MN, USA
[3]Large Lakes Observatory, 2205, E. 5th St., Duluth, MN, USA

[#]Email: schillin@umn.edu

Supplementary Figures legends

Supplementary Figure 1

Expression boxplots of three GH47, four GH76, four GH92 and one GH125-encoding genes differentially expressed (DESeq2; Benjamini-Hochberg (BH) adjusted *P* value < 0.05) across treatments.

Supplementary Figure 2

Expression boxplots of four GH55 **(A)** three GH64 **(B)** and 2 GH71 **(C)**-encoding genes differentially expressed (DESeq2; Benjamini-Hochberg (BH) adjusted *P* value < 0.05) across treatments.

Supplementary Figure 3

Expression boxplots of three GH17 and two GH128-encoding genes differentially expressed (DESeq2; Benjamini-Hochberg (BH) adjusted *P* value < 0.05) across treatments.

Supplementary Figure 4

Expression boxplots of five GH16 and one GH30_2-encoding genes differentially expressed (DESeq2; Benjamini-Hochberg (BH) adjusted *P* value < 0.05) across treatments.

Supplementary Figure 5

Expression boxplots of six GH18-encoding genes differentially expressed (DESeq2; Benjamini-Hochberg (BH) adjusted *P* value < 0.05) across treatments.

Supplementary Figure 6

Expression boxplots of three AA11-encoding genes differentially expressed (DESeq2; Benjamini-Hochberg (BH) adjusted *P* value < 0.05) across treatments.

Supplementary Figure 7

Expression boxplots of one each GH18, GH75, GH89 and GH20-encoding genes differentially expressed (DESeq2; Benjamini-Hochberg (BH) adjusted *P* value < 0.05) across treatments.

Supplementary Figure 8

Expression boxplots of eight protease-encoding genes differentially expressed (DESeq2; Benjamini-Hochberg (BH) adjusted *P* value < 0.05) across treatments.

Supplementary Figure 9

Expression boxplots of three AA1-encoding genes differentially expressed (DESeq2; Benjamini-Hochberg (BH) adjusted *P* value < 0.05) across treatments.

All supplementary figures: Each boxplot represents rlog transformed expression in each sample. Colors of boxplots show carbon substrates (treatments). Median is represented by the box center line, whereas the box represent the interquartile range (IQR).

Supplementary Figure 1

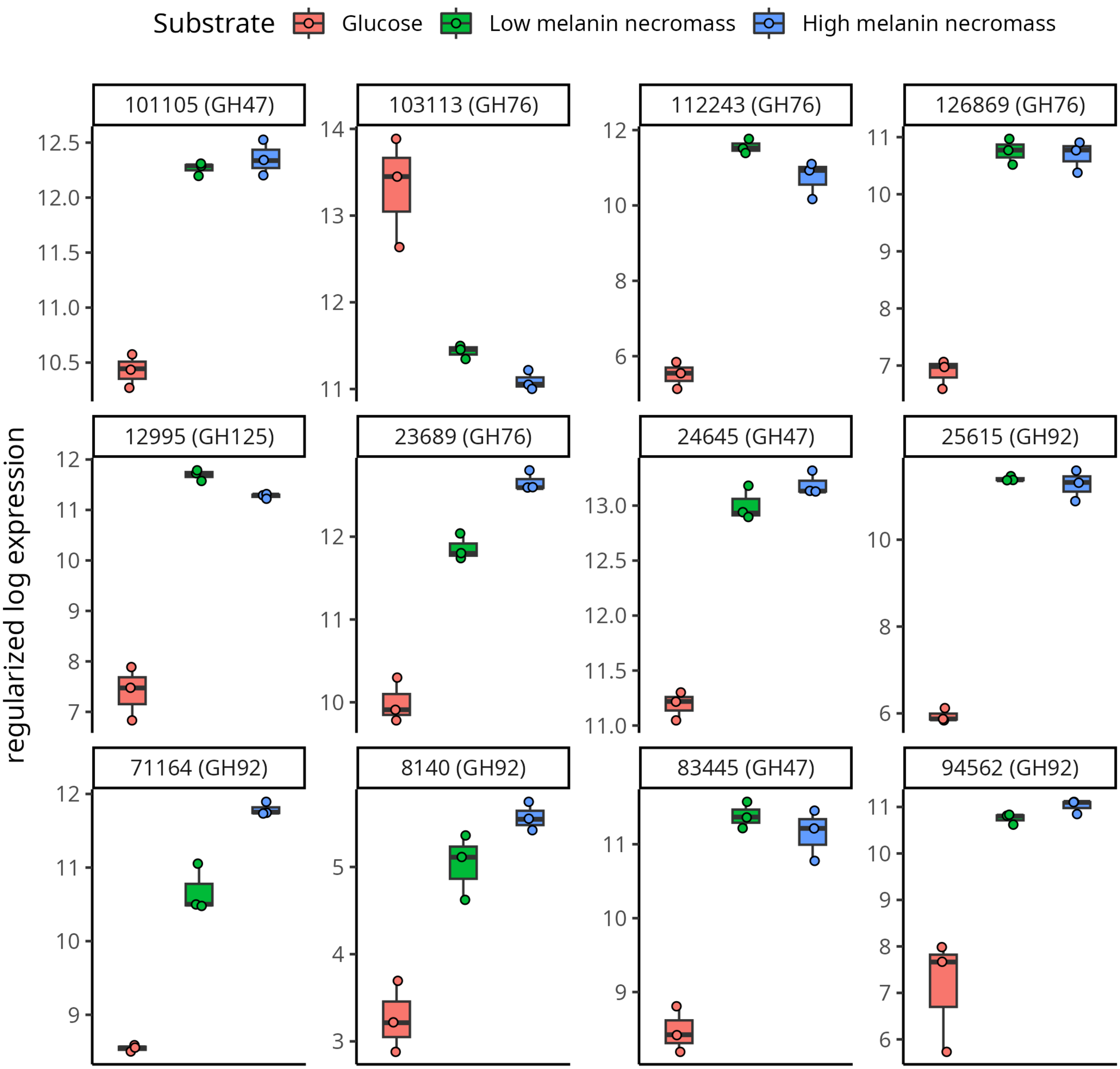

Supplementary Figure 2

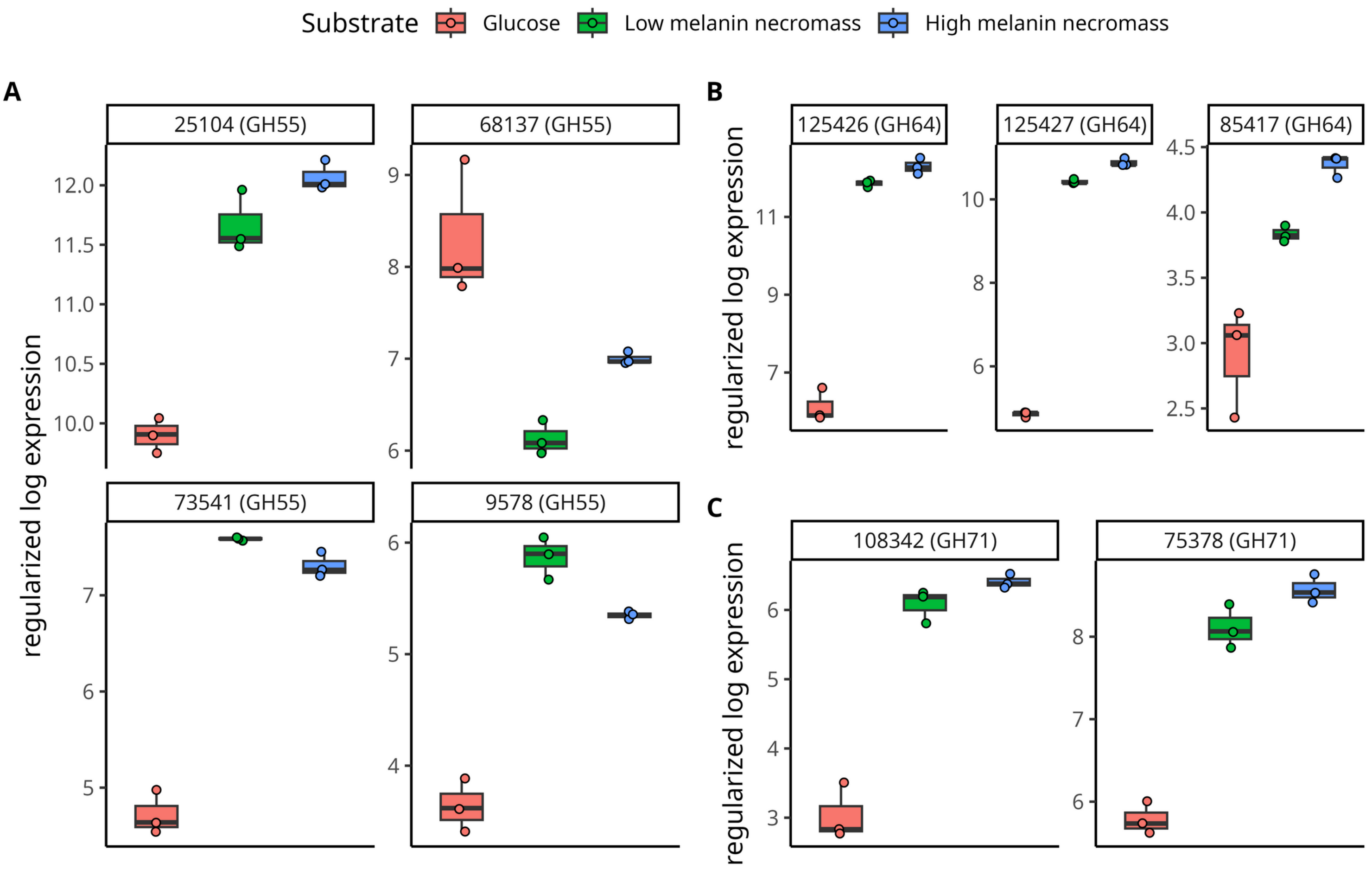

Supplementary Figure 3

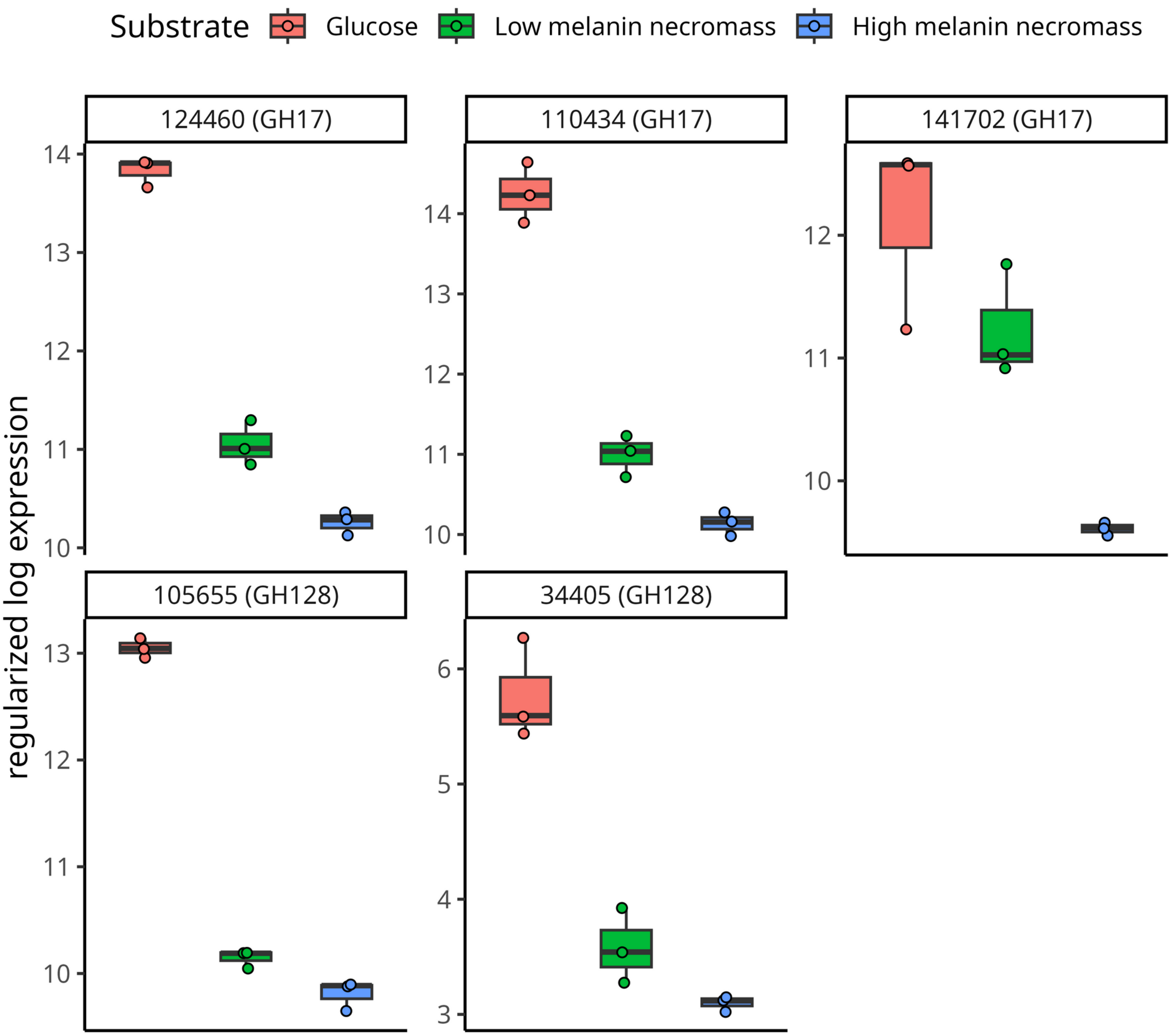

Supplementary Figure 4

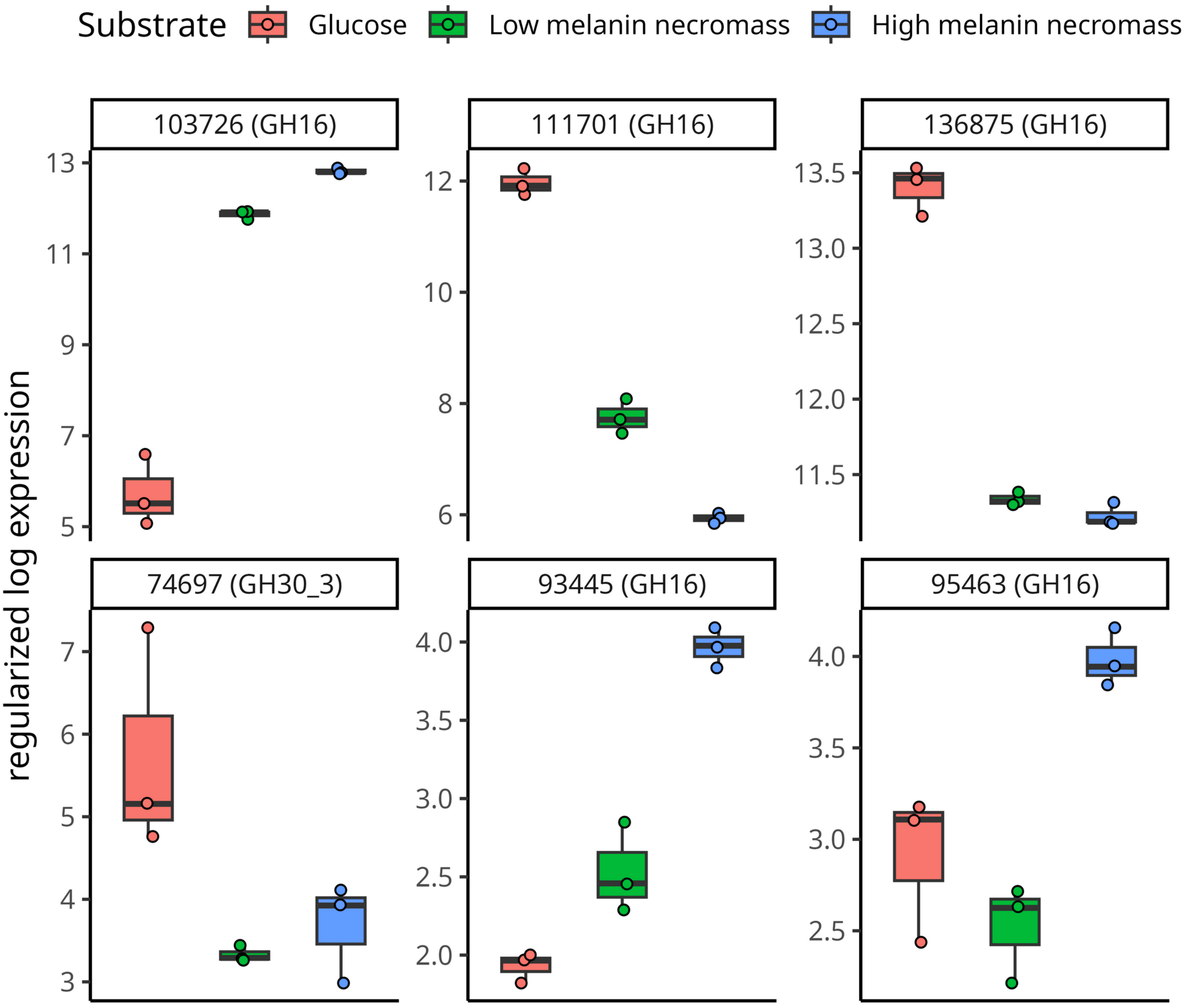

Supplementary Figure 5

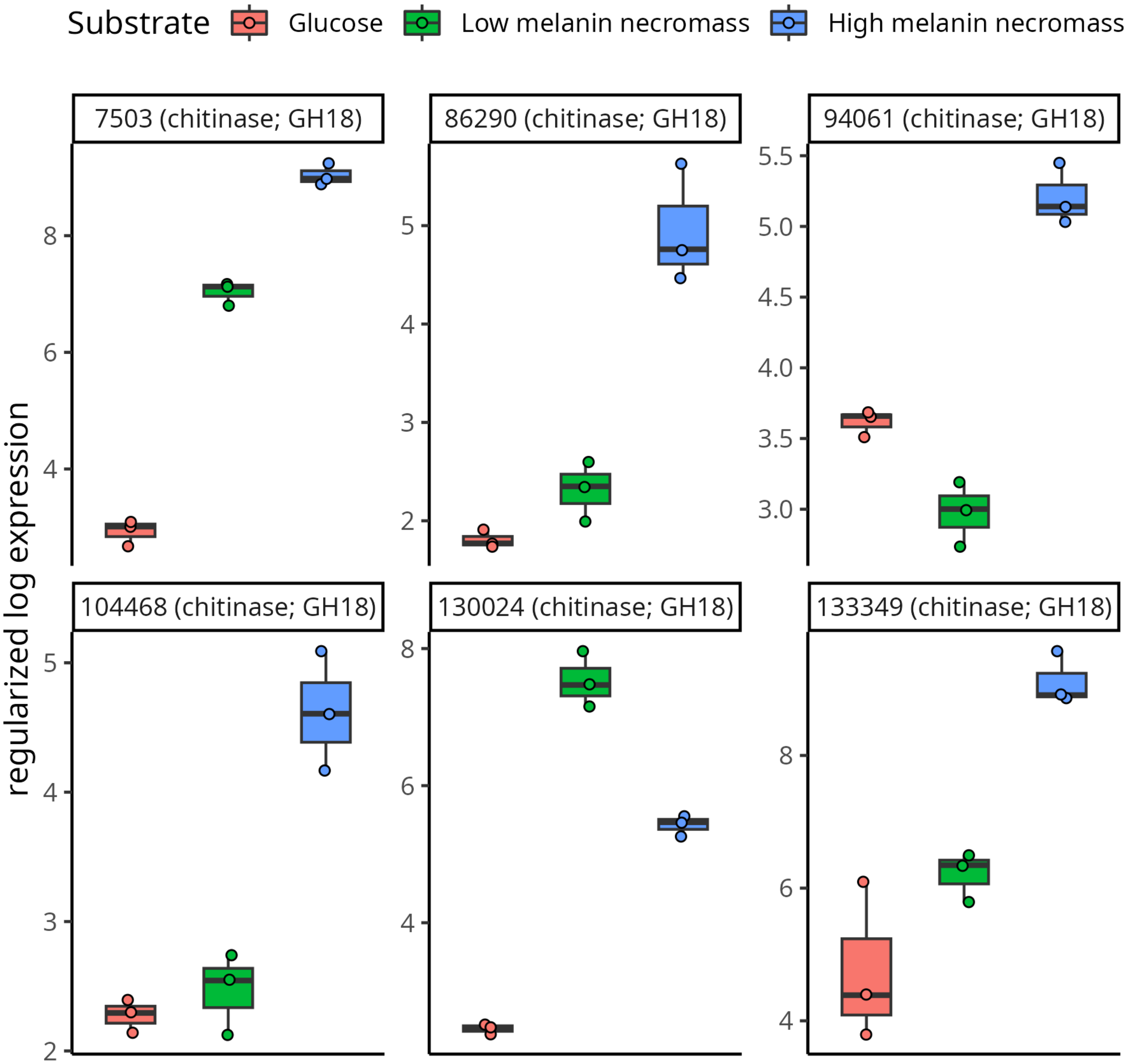

Supplementary Figure 6

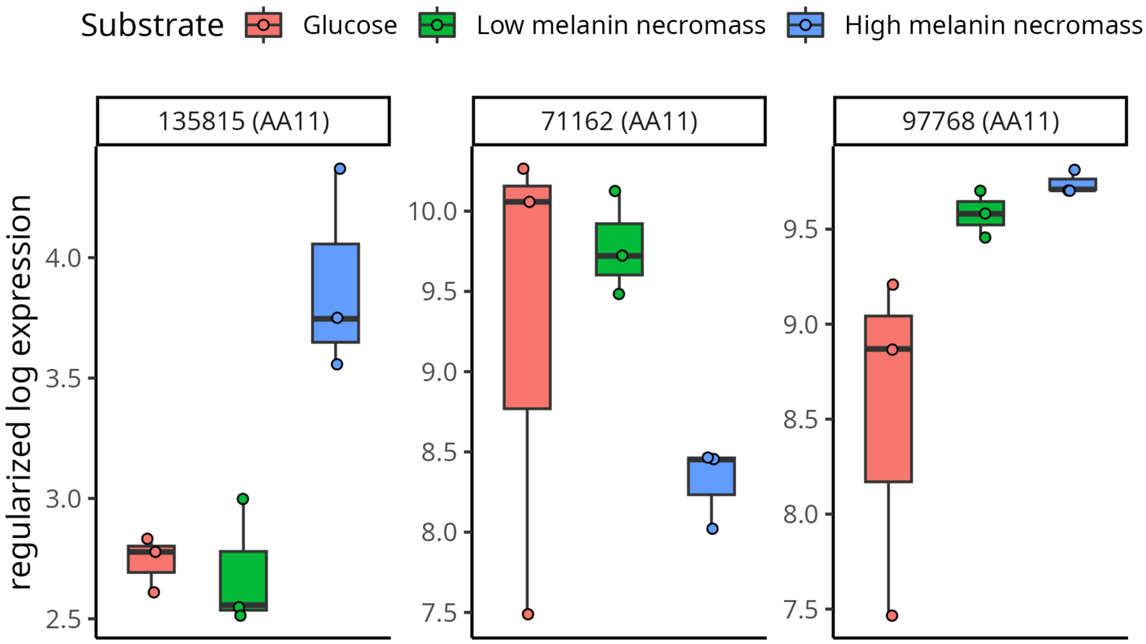

Supplementary Figure 7

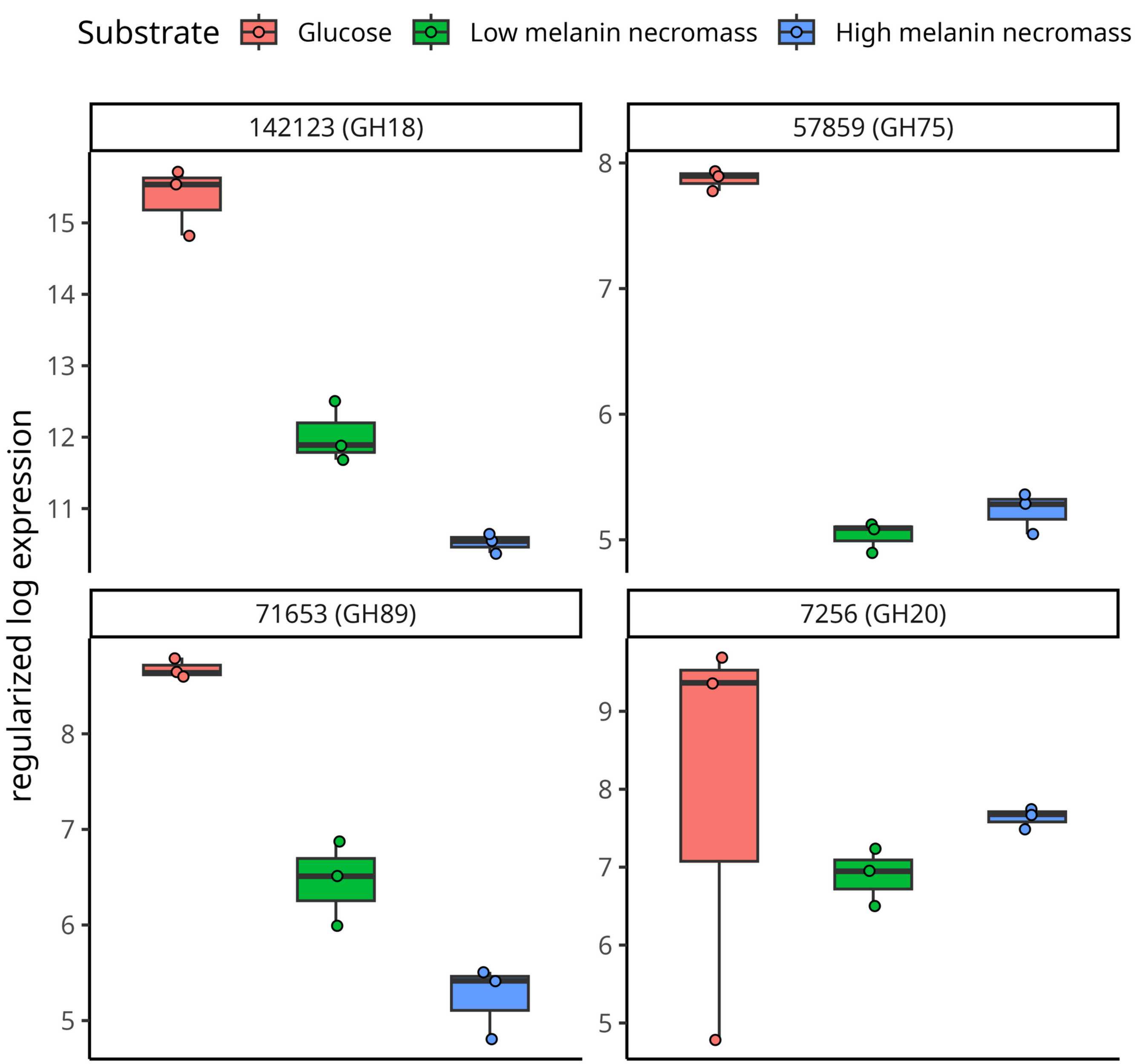

Supplementary Figure 8

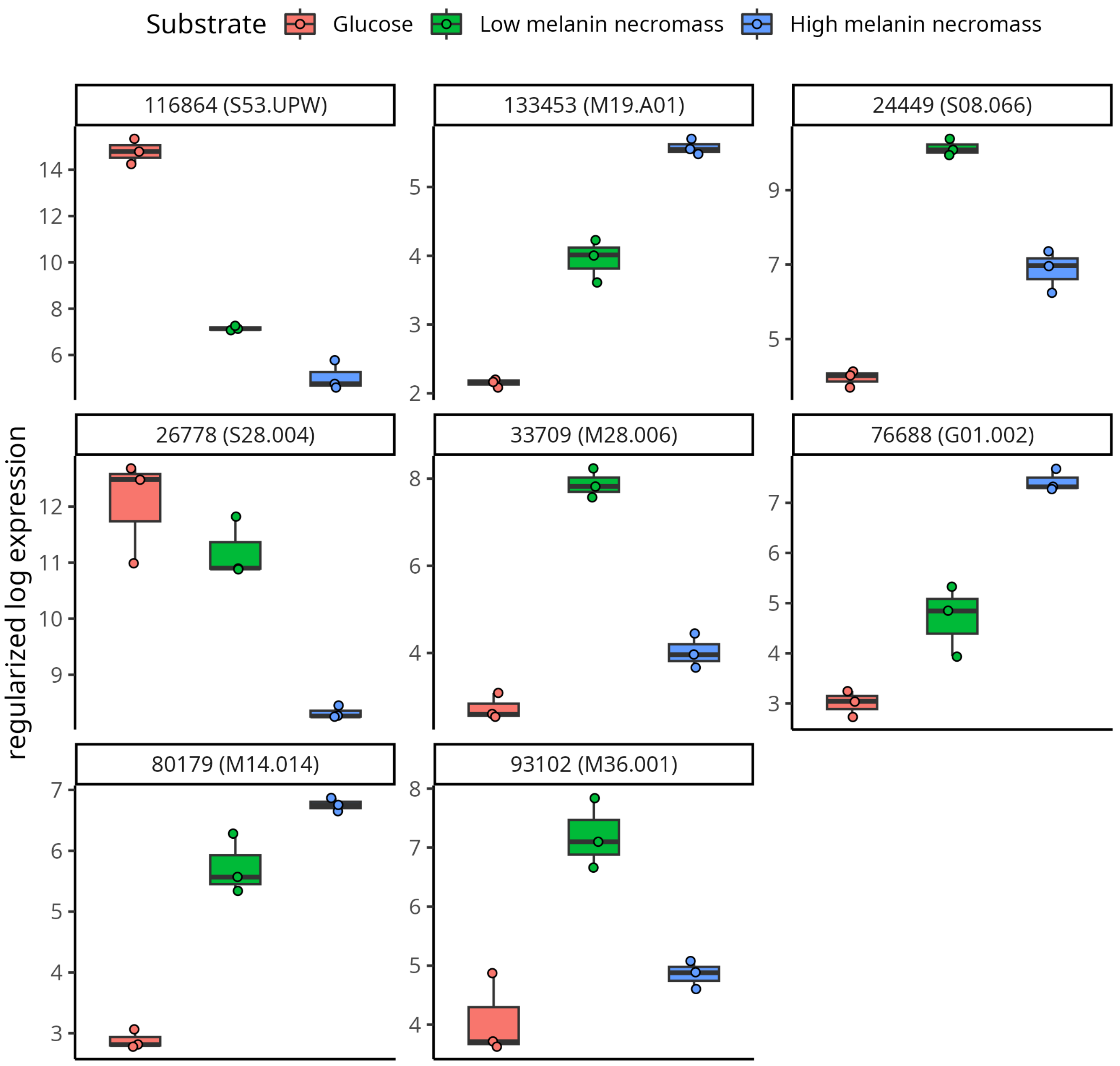

Supplementary Figure 9

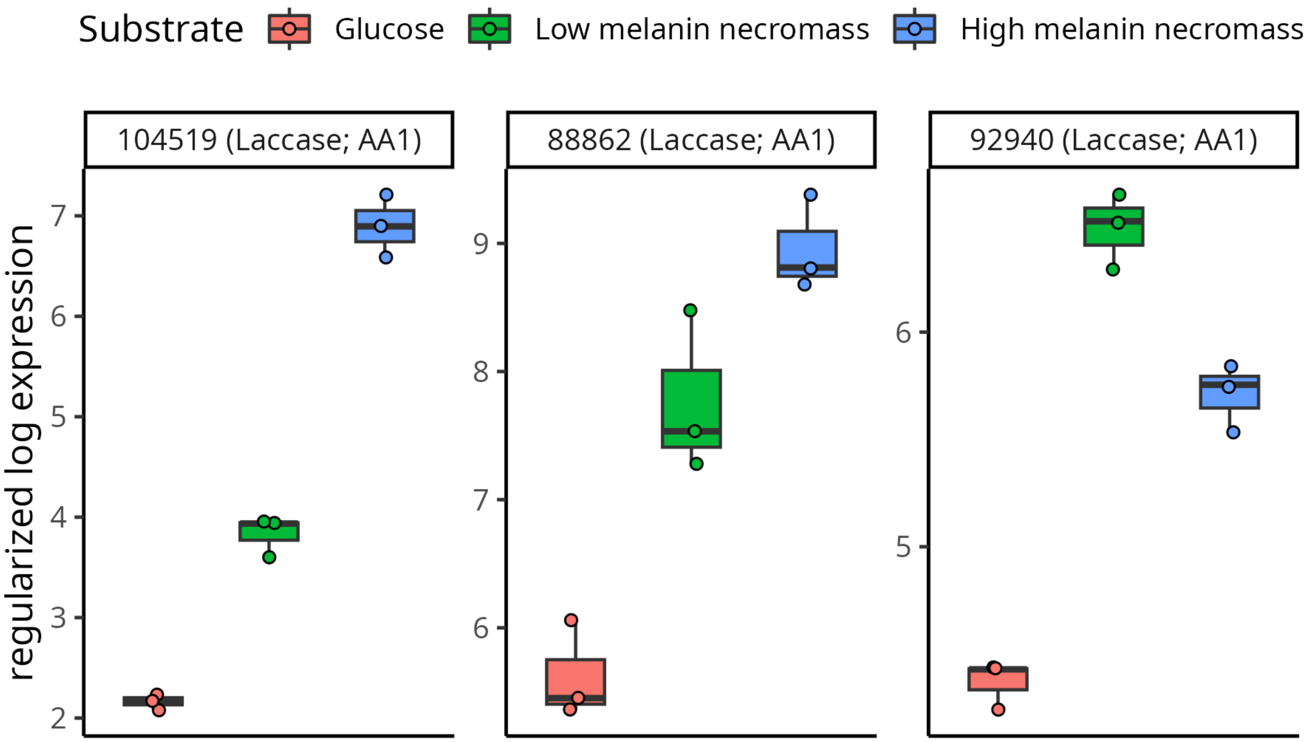

Supplement: Supplementary file 2 — Figure S1. Supplementary Figures. [file EMI-26-e70006-s001.pdf]
